# A qualitative study of child participation in decision-making: Exploring rights-based approaches in pediatric occupational therapy

**DOI:** 10.1371/journal.pone.0260975

**Published:** 2021-12-16

**Authors:** Deirdre O’Connor, Helen Lynch, Bryan Boyle

**Affiliations:** Department of Occupational Science and Occupational Therapy, University College Cork, Cork, Ireland; Imam Abdulrahman Bin Faisal University, SAUDI ARABIA

## Abstract

**Background:**

According to Article 12 of the United Nations Convention on the Rights of the Child, therapists are duty-bound to include children in decisions that impact them. Although occupational therapists champion client-centred, collaborative practice, there remains a paucity of studies detailing children’s rights and experiences of decision-making in pediatric occupational therapy.

**Purpose:**

This qualitative study described the decision-making experiences of children, parents and therapists in occupational therapy.

**Methods:**

Semi-structured interviews were conducted with 17 participants (six children, five parents and six occupational therapists), and data analysed using thematic analysis.

**Findings:**

Three themes emerged: 1) Goal-setting experiences; 2) Adults: child-rights gatekeepers or defenders? and 3) Decision-making in context. Findings suggest that decision-making is mostly adult directed, and children’s voices are subsumed by adult-led services, priorities, and agendas.

**Implications:**

Children’s rights need to be embedded as an aspect of best practice in providing services that are child-centred in occupational therapy practices and education.

## Introduction

Article 12 of the United Nations Convention on the Rights of the Child (1989) asserts that therapists are duty-bound to include children in decisions that impact them [1]. This Article states that governments “shall assure to the child who is capable of forming his or her own views the right to express those views freely in all matters affecting the child, the views of the child being given due weight in accordance with the age and maturity of the child” [1, p.15]. From this rights-based perspective, children’s views cannot be disregarded due to age, or immaturity, yet as per the Committee on the Rights of the Child [CRC] young children and children with disabilities frequently experience barriers to realisation of these rights [2]. For example, within healthcare, child participation in decision-making is uncommon and children’s views are rarely sought [3–5]. Furthermore, this is an under-researched area, particularly in occupational therapy [6]. Subsequently, researchers have advocated for research that examines children’s participation in decision-making in healthcare to understand and address this injustice [7, 8]. For this study, participation is the term adopted in rights-based practice “to describe ongoing processes, which include information-sharing and dialogue between children and adults based on mutual respect, and in which children can learn how their views and those of adults are taken into account and shape the outcome of such processes” [2, p.5]. Frameworks to support participation as an authentic commitment to children’s rights stress the importance of space, voice, audience and influence, in the context of power sharing between children and important adults in their lives [9, 10].

Participation in decision-making is an integral part of the client-centred philosophy in occupational therapy. Client-centredness (which informs child-centred and family-centred practice [11]), embodies a commitment to respect, autonomy and recognition of the clients need for choice in decisions [12]. Yet, Rodger and Keen [11] argue that pediatric practice is often in reality ‘child friendly’ as opposed to child/client-centred. Examples of this child-friendly approach is evident in studies where therapists employ play in therapy but limit the child’s autonomy to selecting toys during intervention [13]. This, however, rarely includes the child’s priorities for play in more significant decision-making contexts for example in goal-setting [13]. This limited autonomy has been noted in other healthcare studies where children are relegated to making small decisions rather than being in the centre of decision-making [14]. These situations arise due to therapists and caregivers typically considering that they have the best interests of the child in mind, and prioritise decision-making on behalf of the child over promotion of their rights [15]. However, children have different views to adults and experience situations differently, which proposes that adult proxies alone cannot give accurate accounts of children’s social worlds or needs [16]. Given this context, it is clear that the therapists commitment to child-centredness is undermined and alternative approaches need to be found.

One way to strengthen child-centred practice is for therapists to adopt the Human Rights-Based Approach [17], or more specifically, a children’s rights-based approach [CRBA] to healthcare where children are considered to be rights holders, and the therapists as defenders of those rights [18]. Within this context of CRBA, therapists are required to move away from a protectionist approach, and to promote child participation in decision-making, “at every level of decision making” [19, p. 9] and “consistent with their evolving capacities” [2, p.23]. Enabling children’s decision-making requires services to implement structures, policy and practices that ensure there is space and time to involve children in their healthcare, including professional training in implementing a CRBA [2]. Such implementation includes provision of appropriate explanatory information relating to the therapy interventions planned, and potential outcomes of such interventions. This serves to ensure that children understand healthcare processes, can express their voice, can make informed decisions, and their views can be given due weight [9]. To date, there is little evidence of the use of a CRBA approach with children among pediatric occupational therapists. Rather, children are considered in the context of a ‘family’ comprising multiple, decision-making stakeholders [20]. In family-centred practice greater emphasis is often placed on adult family members as key stakeholders with children explicitly excluded [20]. Researchers have found that therapists typically give precedence to decision-making with the caregiver (rather than the child) [21]. Although such practices may appear in the best interest of the child, instead the child’s right to express their views is denied. While caregivers should be closely involved in decision-making on behalf of the child, nonetheless valuing caregivers’ opinions over the child’s dominates many healthcare studies, resulting in the need to consider where has child-centred practice gone? [14].

In occupational therapy studies exploring children’s rights and their perspectives on decision-making [6, 22], children identified experiences of being excluded from decision-making, and being ill-informed despite therapist’s efforts to explain and dialogue with them. Even when they felt able to express their opinions, they reported that adults listened but did not take their views into account when making decisions [6]. Consistently, children in these studies reported that they valued being involved in decision-making and gave examples of decisions they made in other contexts; yet in the therapy context, they deferred to the adult often due to poorly established child-centred approaches to power-sharing and empowerment. The findings are similar to those from other healthcare studies, where it has been documented that attempts made by children to participate are “often thwarted by adult’s actions” [7, p. 1687]. Such examples identify healthcare contexts whereby few efforts are made to provide space and time for children to express informed views, with even less effort on hearing the children’s views, and being influenced by them which according to Lundy, is fundamental in implementing rights-based approaches [9].

Decision-making in pediatric rehabilitation is especially evident in goal-setting processes where collaborative dialogue and power sharing occurs [6], and during which clients and professionals make decisions and agree on goals [23]. From a scoping review of 62 studies of goal-setting in children’s rehabilitation, researchers identified that carers and teachers have been the main stakeholders, while the conspicuous absence of children in the process was noted [24]. While some evidence shows positive outcomes for goal attainment, and increased motivation of the child when child-centred goal-setting is implemented, conversely also the lack of child determined goals was noted as possibly contributing to a lack of motivation to achieve goals [24, 25]. Challenges to collaborative goal-setting in pediatric occupational therapy include the “abstract” process of identifying and prioritising goals [25], the diversity of opinions and concerns by therapists of families proposing unrealistic goals [24], and difficulties in communicating health information in a way for parents to understand [21]. These challenges are amplified by the power imbalances that frequently exist regarding decision-making authority between children, parents, and occupational therapists [21]. Indeed, Kramer et al. [26] found that despite therapists’ commitment to the ideas of child-centredness, professional knowledge was prioritised over the knowledge of children and parents during decision-making in goal-setting.

In summary, within occupational therapy, child decision-making underpins client-centred practice and in goal-setting specifically. However, few studies to date have explored the child’s role in decision-making in this process. Some recent studies have focused on goal-setting practices within pediatric occupational therapy but examined aspects such as therapists perspectives [27, 28] and the process of determining goals [29] without examining decision-making from a child’s perspective. Furthermore, in their scoping review of goal-setting research, Pritchard-Wiart and Phelan [24] identified that no qualitative studies to date have been conducted with children in pediatric occupational therapy, suggesting the need to examine the child’s perspectives of the goal-setting process. The same authors have subsequently worked to address this gap in more recent work whereby children’s experiences were documented to understand goal setting as a co-constructed process [30]. Therefore, the complex nature of collaborative intervention planning and provision in triadic relationships requires consideration of multiple perspectives of professionals, parents, and children in understanding children’s participation in decision-making in general, and in goal setting in particular [7, 26].

Thus, to address this knowledge gap, the aim of this study was to describe perspectives and experiences of children, parents, and occupational therapists concerning children’s participation in decision-making in the therapy process, including goal-setting, as well as examining the contextual influences to this process. The research questions were as follows: 1) How have children, parents and occupational therapists described children’s participation in decision-making within the goal-setting process? and 2) What are the contextual factors that influence children’s participation in decision-making in therapy process?

## Method

### Design

This study used a qualitative description design [31, 32] to describe the perspectives and experiences of children, their parents and occupational therapists concerning children’s participation in decision-making in the occupational therapy process. Qualitative description is commonly used to examine health care phenomena and is appropriate for research questions that focus on exploring the who, what and where of experiences that goes beyond basic description, in order to gain a “rich, straight description of an experience” [33, p. 2]. It is used to understand a process and participant perspectives [32]; thus clearly aligns with the aim of this study to describe the experience of participation in decision-making processes from the perspective of those directly involved in the process. Participant views are central from an occupational therapy lens, as the profession values and respects client narrative and autonomy, thus aligned with the approach.

Qualitative description design is beneficial in health science research as it offers important information for clinical practice [31] and insights for practice improvement [33].This approach draws from ontological and epistemological positions of interpretivism and constructivism. From a constructivist perspective, the research was grounded in valuing the participant’s views and experiences [34]. Utilising an interpretive approach, researchers recognised their influence on the process in that qualitative research is a co- construction of knowledge between the researcher and the participant. Ethical approval was obtained for this study from the Social Ethics Research Committee in University College Cork in January 2019 (Log 2018–203).

### Recruitment

To be eligible for participation in this study, child/adult inclusion criteria were: (i) a minimum of one child and one adult (who typically accompany the child/young person to occupational therapy, e.g., parent, guardian, or caregiver) and (ii) the child was currently attending or has attended a complete course of occupational therapy in the past. Occupational therapy was determined to include goal-setting as a composite part of the process. Occupational therapists were required to have at least one year of experience of working in a pediatric setting. Informed consent from occupational therapists, and both child assent and parental consent were required (see Table 1 for inclusion criteria). Purposive sampling was utilised to recruit child-parent units and occupational therapists from the Munster region of Ireland with the assistance of two gatekeepers. Snowball sampling was adopted to recruit therapists after a period of purposive sampling stalled and an alternative approach was required. Recruitment was open to any child (under 18 years) and family who were referred to occupational therapy services with occupational challenges in school participation, for example handwriting. These occupational therapy services were clinic based and provided community services to children with neurodevelopmental disorders who were attending mainstream schools in the region. For the purpose of this study, the UNCRC [1] definition of children (those under 18 years old) was utilised, and diagnostic criteria was not considered in participant selection in line with the human rights model and the social model of disability. All prospective participants were provided with information about the study by email and postal letters (which included information about their right to withdraw, for example). The first child-parent units and therapists to consent to take part were accepted for the study.

**Table 1:** Inclusion criteria for child-adult units and occupational therapists.

Child-adult unit inclusion criteria	Occupational therapist inclusion criteria
1. Each child-adult unit consists of a minimum of one child and one adult (who typically accompany the child/young person to occupational therapy, e.g., parent, guardian, or caregiver);	1. Occupational therapists were required to have a minimum of 1 years’ experience working in a pediatric setting to ensure they were familiar with processes and procedures in a pediatric context; and
	2. informed consent was obtained.
2. the child was currently attending or has attended a complete course of occupational therapy (including evaluation, intervention, targeting of outcomes) in the past; and	
3. both child assent and parental consent was obtained.	

### Participants

17 participants (six children, five parents and six occupational therapists) were recruited. Written informed consent was obtained from parent participants for their child’s participation as well as obtaining the child’s assent. Obtaining assent is not a legal requirement, however, it reflects the significance of the child’s voice and is in line with best practice guidelines in Ireland developed by the Department of Children and Youth Affairs [35]. All six children were able to understand the research and assented to take part independently from their parents. In total, 11 dyads of child-parent participants (i.e. five family units consisting of six children and five parents) offered informed consent/assent to participate in this study. Of note, one parent had two children attending occupational therapy who participated in the study, hence the imbalance of participant numbers in the study. Three children had attended/were attending public therapy services (i.e. government provided) and three children, private services (i.e. self-funded) (Table 2). All were attending for concerns regarding motor challenges impacting their school occupations.

**Table 2:** Child and parent participants.

Child	Age of the child	Parent of the child	Type of Service Accessed (Public/Private OT)
**Emily**	6 years and 4 months	P1 Emily’s parent	Public: Early Intervention (EI)* (Had attended private services previously)
**Tom**	12 years and 2 months	P1 Tom’s parent	Public: School Aged (SA)**
**Jason**	11 years and 10 months	P2 Jason’s parent	Private***
**Jack**	10 years 11 months	P3 Jacks’ parent	Private
**Eddie**	9 years and 11 months	P4 Eddie’s parent	Public: SA
**Bill**	8 years and 3 months	P5 Bill’s parent	Private

Notes

* In Ireland, children from 0–5 years attend EI services.

** In Ireland, children from 5–18 attend SA services.

*** In Ireland, private services normally have clients from 0–18 years old.

Six occupational therapists consented to take part and written informed consent was also obtained from them. All six recruited therapists were working in public healthcare services (i.e., government funded) (Table 3). Although the goal was to recruit triads of child-parent-therapist, this proved to be unattainable during the implementation phase of the study, and therefore the first six therapists who consented to take part were recruited; the final six participating therapists were not delivering services to the participant families. Overall, 17 participants (six children, five parents and six occupational therapists) were included in this study. A defined sample size was not determined from the outset and the decision on sample size was based upon satisfactory saturation of ideas which was deemed to have been achieved upon completion of 17 interviews.

**Table 3:** Occupational Therapist (OT) participants.

OT	Service	Years’ experience as an OT
**OT1**	Public–Early Intervention Service [EI]	1.5 years
**OT2**	Public–EI	7 years
**OT3**	Public -School aged service [SA]	3.5 years
**OT4**	Public–SA	15 years
**OT5**	Public–SA	1.5 years
**OT6**	Public– 0–18	30 years

### Data generation

The views and experiences of children, parents and occupational therapists were obtained using semi-structured interviews, which included questions informed by literature. Interviews are a popular method of data collection in qualitative descriptive research as they are useful in gathering information that describes the experience from participant’s viewpoint [36] and thus were relevant to the research questions. However, the approach to researching with children needs to be considered carefully, to ensure the devolution of power from researcher to participant [35]. For example, the process of interviewing children can be enhanced by using a range of customizable interview techniques which support a dynamic, and individualised interview method [37]. In this study, each research encounter with a child was approached with a toolkit of strategies including 1) *drawing* [38], 2) *role-play* [39], and 3) *photo elicitation* [40] to facilitate children’s ‘voice’ to be conveyed through a variety of media of the child’s choice (as per the UNCRC [1]) rather than relying exclusively on written or spoken language.

In consideration of the need to equalise power relations between this researcher and participating children, interviews were conducted in family homes (parent and children) during April and May 2019. Child and parent semi-structured interviews took place on the same day. Parents were invited to be present while the researcher was completing data collection with the child, as required by the Ethics Approval Committee. Awareness about how this parental presence may facilitate and/or hinder a child’s views being heard was an important consideration for the researcher, however [41]. To mitigate possible impact, efforts were made by the researcher to create an informal atmosphere by adopting a casual dress code, communicating at eye level, and utilising language appropriate to the age of each participating child. The researcher provided assurance to the children that they did not have to answer or do anything that they did not want to and that there were no right or wrong answers to the questions that were asked [42]. Role play and drawing were used informally to build rapport with children immediately in advance of conducting each interview. Despite being offered the aforementioned media to communicate their voice, all child participants chose to communicate verbally with the researcher (i.e., through a semi-structured interview). Picture elicitation was used and involved showing a child pictures of typical pediatric occupational therapy clinics to elicit discussion about their experiences. Occupational therapist interviews were completed either in the clinic space in which they worked in or over the phone. All participants participated in one interview each, resulting in a total of 17 semi-structured interviews being completed.

### Data analysis, trustworthiness and methodological rigor

Interview data obtained from children, parents and occupational therapists were audio-recorded and transcribed, and analysed by the researchers [DOC, HL, BB] using Braun and Clarke’s (2006) six-step approach to thematic analysis (*becoming familiar with the data*, *generating initial codes*, *searching for themes*, *reviewing themes*, *defining themes*, *and writing up*) [43]. As drawings were used only to build rapport through the choice of the child participants themselves, it was not formally analyzed by the researchers. To become familiar with the data, it was read and re-read by the researchers. The data were coded manually which generated reflective notes that were utilized to begin the categorization of the data. The first author generated initial codes from the interview data. These codes were then reviewed by the second and third authors and these codes were collated into themes. The themes were confirmed among the three authors. Initial codes, and themes were derived from the data (i.e. not identified prior to analysis), and the process of generation, review and defining was led by the main author in collaboration with the co-authors to ensure rigour and dependability [44]. Peer debriefing, as well as maintaining meeting minutes and decisions regarding coding ensured an audit trail. To enhance credibility, member checking was used with participants to give them the opportunity to verify or challenge data accuracy and interpretation [44]. A member check interview using the transcript of the first interview was completed with children while transcribed data was returned to adult participants to ensure accuracy of transcribed data [45, 46]. As the researchers are occupational therapists it was critical to participate in reflexivity to examine how the researchers background and perceptions may impact the research. For example, researchers reflected in journals on their background and how/if this may have impacted on the willingness of participants to talk freely about their experiences. The researchers remained aware of their role in influencing interpretation throughout the analysis. Further, openness about the ontology and epistemology held by the researcher adds reflexivity.

## Findings

The analysis resulted in three major themes with six associated subthemes (Table 4). Theme one described participants experiences of decision-making and goal-setting. Theme two examines power and autonomy within this decision-making process, while theme three situates decision-making in context of practice. Participants’ identities have been protected in the presentation of findings using pseudonyms and study numbers.

**Table 4:** Themes and subthemes.

Themes	Goal-setting experiences	Adults: Child rights gatekeepers or defenders?	Decision-making in context
**Subthemes**	• The who, how and what of goal-setting in pediatric occupational therapy	• Knowledge, views, and attitudes	• The “fight” for services
			• Aspirations and Actuality
		• Power and influence in pediatric occupational therapy	
	• Decision-making: Shared or restricted?		

### Theme 1: Goal-setting experiences

The first theme details the participant’s experiences of decision-making through the lens of examining the process of goal-setting in occupational therapy as well as the power-sharing practices that were experienced.

#### The *who*, *what* and *how* of goal-setting in occupational therapy

Occupational therapists described the process of goal-setting as “informal” (OT1), “non-standardised” (OT2) and as a “discussion” (OT3) whereby information from assessment, parents, school and teams “concerns and priorities … inform goals” (OT1; 0–5 service). Although standardised tools were mentioned (e.g., Perceived Efficacy and Goal-Setting System [PEGS] and the Canadian Occupational Performance Measure [COPM]), most reported not using them currently. So, from the therapists’ perspective, goal-setting was an informal process that involved information-sharing, dialogue and shared decision-making. However, whether the child was included or not seemed largely dependent on the age of the child:

“Generally, it would be myself and the parent … it would be from the very beginning involving parents in what their concerns are what are their priorities for treatment for OT, then what my priorities the team’s priorities are….” (OT1; 0–5 service)

While the child’s voice was missing in this process for younger children, occupational therapists working in a school aged service (6–18 years) stated that older children “are a huge part of the goal-setting process” (OT5). Asking a child about “what [their] issues were” (Jack’s parent) seemed common. Occupational therapists interpreted what children stated in forming goals: “… [a child might say] I get so angry and I don’t know how to help myself … me and the parent … [take] that as an expression for help … it does still come from listening to the child … [but] we’re kind of formulating … [their goal]” (OT3).

Yet, half of the children felt they were not involved in the goal-setting process, with Tom, Emily, and Eddie all answering “no” when asked if they felt that age matters in relation to being part of decision-making in goal-setting.

One child reported that not being included made her feel unhappy “she made me feel sad … Very very sad” (Emily). The other three children felt included in this process, with one parent stating that “she [the OT] listened to the child” (Jason’s parent). However, some parents acknowledged their child’s non-involvement in goal-setting:

“Handwriting was … always a target for [Tom] … Now he I suppose it was explained to him that look you need to start typing because you’re finding handwriting quite difficult. He bought into that so perhaps that just why he did better, but no he was never … asked” (Tom’s parent).

Ultimately, parents asserted that goal-setting should be “unique to the child” (Emily and Tom’s parent). Therapists also asserted this need for tailoring to the child’s needs, which relied on their perceptions of a child’s ability to engage with the information and also the impact that the information might have on the child. For example, one therapist noted the need to protect certain children from information, and used the example of a child presenting with anxiety:

“… depends on the child. I had a child recently he was … really anxious. So then there was no way I was going to say anything about assessment … because I could tell it was going to make him more anxious… “(OT6)

Overall, while occupational therapists spoke of collaborative goal-setting generally, it seemed that occupational therapists “rely heavily on what the parents say …[as] they know their children best” (OT6). Furthermore, despite knowing that client-centred practice includes the voice of the child, parents’ views were more typically prioritised, and it was unclear the extent to which children felt empowered or listened to during the process of goal setting.

#### Decision-making in the goal-setting process: Shared or restricted?

Occupational therapists felt that they are “guiding” (OT1) goal-setting, ultimately, holding significant power, with one child stating that the “bossy one [referring to the OT]” chose her goals in occupational therapy: “No, they just say now you have to do this, you’re not allowed to do that” (Emily). One occupational therapist spoke of each member of the triadic relationship having a goal for occupational therapy:

“For goal setting I’ll often try to get the child to pick up one goal and maybe the parent pick a goal and then I’ll pick a goal as well if I feel like we are capturing everything that we need to work on” (OT3; 6–18 years’ service)

When goals of the child differed from goals of the parent, for example, “handwriting” (OT3), occupational therapists described their role as “mediator” (OT4) for collaborative goal-setting to ensure everyone is satisfied: “You’ve got one you want. He’s got one he wants, and I’ll take the rest” (OT4). Further, occupational therapists acknowledged that children are “not going to want to do every single goal that you pick out for them [the children]” (OT3).

Finally, therapists described their role as “orchestrator” of power-sharing during therapy. For example, where children had to do certain activities (e.g., take part in a handwriting group), occupational therapists stated that they would always incorporate the child’s interests and allow the child to make small decisions “… a little boy is obsessed with football so [we write] about [a] favourite football player” (OT3). Decision-making was more apparent in other ways (aside from goal-setting). For example, parents and occupational therapists stated that children may be involved in decisions when offered specific choices “[children] would have a choice over what they might do in the session” (OT2). In such cases, therapists would have selected activities for the child to complete and the child chooses one activity “we’re doing one of the 3 [activities] but which one do you want to do (P5)”.

However, some parents reported therapists chose the goals primarily “… [goals were] always decided for [the child] by the occupational therapist” (Emily and Tom’s parent). Parents felt therapists were “expert” (Bill’s parent). Parents valued collaboration “children should have a voice… but … we need to be led by the experts … [however] there needs to be collaboration …” (Bill’s parent). Furthermore, this parent recognised the need to share power in the goal-setting process but identified “I’m not sure what that looks like” (Bill’s parent). Children in this study also agreed that children have to do things they may not want to do in occupational therapy, however, they argued for a balance: “you probably should have free choice for a little bit …” (Bill), “we should be doing the therapy but we should also be having fun at the same time” (Tom). So, while both children and parents valued power-sharing for the child, they lacked certainty on what that meant for decision-making in the therapy context.

Occupational therapists felt that negotiated decision-making in goal-setting that included the child’s voice had a positive impact on children’s attainment of goals. One therapist stated “… this actually worked when I took time to actually listen to the child and pick out their goals” (OT3). Occupational therapists reflected on their own power in practice, however, with one asking: “Did I actually consider what the child wanted?” (OT3). Furthermore, expectations of adults for children had one occupational therapist reflecting on goal ownership: “… if you’re referred in from school and they’re worried about handwriting … whose goal is that? Is it the parents? Is the child’s? Is it the school? Is it the system?” (OT6). Significantly, this occupational therapist identified the complexity of goal setting, and questioned: whose goal is it anyway?

### Theme 2: Adults: Child rights gatekeepers or defenders?

This second theme details children’s, parents’, and occupational therapists’ awareness of the United Nations Convention on the Rights of the Child (1), and the rights of the child, as well of their views and attitudes concerning children’s participation in decision-making and consists of two subthemes.

#### Knowledge, views and attitudes and their subsequent impact on children’s participation

Children, parents, and therapists were not aware of the existence of Article 12 of the United Nations Convention on the Rights of the Child [1]. Yet, children, parents and occupational therapists felt it is “very important” (Jack) for children to be included in decision-making, especially around the goal-setting process, “because it affects them 100 percent” (Jack’s parent), and so that they are “encouraged or motivated by the goal” (OT1) (OT5). Still, children identified the need for adult involvement because “adults know more than kids” (Bill).

Power sharing was highly dependent on the child’s age and abilities according to both therapists and parents, as opposed to being viewed as a right of the child. Occupational therapists asserted that a younger child (under 5) may find it “challenging … to come up with a specific goal” (OT2) and “require support from their parents” (OT3). Further factors such as a child’s “poor attention/concentration” (OT5), “anxiety” (Bill’s parent), perceived low “self-esteem” (Jack’s parent) and a “child’s mental health” (OT6) impacted their participation in decision-making, as there were communication challenges. Therapists stated that children living with “intellectual disabilities … [and] speech/language difficulties” (OT1) are “not really heard” (OT4) and “they aren’t as included in decision-making [at the same level] as a child with normal cognitive ability or with no learning difficulties” (OT1). This is despite therapists’ awareness of the importance of “the child’s voice [being] embedded … in [occupational therapists] training and in [occupational therapy services]” (OT3). Nevertheless, one therapist noted that “they still have a voice” (OT4).

Both children and parents identified that children’s participation was impacted by the occupational therapist’s ability to ask and listen to what children had to say. One child identified that the best occupational therapist they had encountered while availing of occupational therapy services was someone who allowed him to communicate what he wanted to talk about: “probably the best … because I’m allowed to talk” (Tom). The importance of this was reiterated by occupational therapists who acknowledged that “respecting them … listening to them [the children]” (OT3) and having “good therapeutic rapport” (OT1) facilitates their participation in decision-making. Other factors that facilitated participation for these children included becoming familiar with the child and the use of visual aids to help explain things. Occupational therapists accepted that their own abilities impacted the child’s voice and participants offered numerous suggestions of other facilitators to children’s participation including training, liaising with other professionals, and an effective therapeutic relationship, for example.

Overall, although children’s rights were valued by all, knowledge about the centrality of the child’s right to being heard and to participate in decision-making was absent; instead, occupational therapy practice was informed by client-centred philosophies which were taught in professional training.

#### Power and influence in pediatric occupational therapy

Power and influence were highly related to doing what the adult’s thought was best for the child. Parents discussed having to make decisions for their child’s “own good” (Emily and Tom’s parent). One child was aware of this issue and gave an example of not having a choice to stop attending an occupational therapist in a school setting: “my mom just wants me to keep doing it… but if I had a choice, yes, I would stop …” (Tom).

Nonetheless, there were also examples of enabling participation in power-sharing in the child-adult relationship. Parents expressed how they listened to their child when the child voiced their opinion: “[Eddie] doesn’t like the exercise that the physio [and OT] gave him; they were really strenuous …. he hated it … so we stopped making him do it” (Eddie’s parent). In another instance, an occupational therapist recognised that the child’s agenda was different to her own from a therapy perspective and realised the importance of hearing the child’s voice:

“… we had a little boy come in recently and he just didn’t want to do anything. and mom was really forcing him to kind of perform. And I just said look there’s no expectation here. If we get something, we get something if we don’t, we don’t if he doesn’t want to partake that’s up to him as well that’s fine. He probably doesn’t feel comfortable about it. So … mom and I chatted about general things … and then we kind of got some games out and I let him, and mum participate with that and then I asked him was it OK if I joined in. And then he gave me permission. So, you can’t force a child if they’re not ready or willing and so you need like OK that was my agenda” (OT6).

This therapist was clearly concerned with the child’s motivation, autonomy, and the need for mutual commitment to the therapy process.

In summary, power and influence was evident in many ways, when adults listened to the child, engaged in a process of power-sharing and shared decision-making, that sometimes resulted in the child influencing the outcome.

### Theme 3: Decision-making in context

The third theme describes the experiences of pediatric occupational therapy in the Irish context from the perspectives of children, parents and occupational therapists and involves two subthemes concerning accessing services, and realities of practice. Findings about the broader context are presented, as experiences of decision-making cannot be understood in isolation from the institutional context in which it occurs.

#### The “fight” for services

Both parents and occupational therapists spoke about issues with accessing services, for example, the waitlist [for public services] “is three years” (OT3); “people have to fight for so long to get [services]” (Jason’s parent). There was a perceived need to attend a “private OT” (Eddie’s parent) due to waiting lists; however, both children and parents spoke of the financial implications of this: “700 euro [for assessment and intervention]” (Eddie’s parent); “my mom had to pay too much money … I had to stop” (Emily). Occupational therapists acknowledged parent’s frustrations and referred to waiting lists:

“the waiting [list] … is three years so by the time the parents see us you know they’re understandably very frustrated” (OT3)

When assessment was received parents reported further barriers to service delivery. For example, one of the children had assessment but had no occupational therapy intervention due to further waiting lists for public occupational therapy intervention services:

“A lot of leaflets but no actual intervention… No nothing for eighteen months. Welcome to Ireland” (Eddie’s parent).

Furthermore, it was clear that with delays between assessment and intervention, parents often felt they had no other option but to conduct home programmes based on outdated reports:

“I don’t even know what he needs anymore … I don’t even know do I still need to be walking him around like a wheelbarrow” (Eddie’s parent).

All occupational therapists felt that “public services are under-resourced in terms of staff” (OT1), and accordingly felt pressure to run groups “to get through people [on the waiting list]” (OT5) which made it “very hard to individualize goals” (OT5). Given these issues, an occupational therapist stated: “… sometimes you rush in [and identify] these are the things we’re going to work on and thinking [and later question] have I actually asked the child if he wants to work on these things?” (OT 3). Because of the perceived barriers, particularly within the public system, occupational therapists felt “that goal setting can … slide down the importance list” (OT3).

#### Aspirations and actuality

Despite a fight for services, occupational therapists reported that working in a family-centred manner was important, but rather than it being a reality, instead it was still something to “aspire to” (OT6). Therapists reported “accommodating parents with appointment times” (OT5) and obtaining “feedback from parents …[on] how to improve services” (OT1) as a way to operationalise family centred practice. Therapists noted many things that they regarded as not being family centred, for example service locations and having limited opportunities to liaise with other professionals.

Family’s experiences of family-centred practice also highlighted some limitations. Parents described being “clueless about occupational therapy” (Bill’s parent) as well as being unsure of “how OT was going to help” (Eddie’s parent) yet had expectations “people think … I’ll bring my child to this appointment for an hour and it will solve everything” (Jason’s parent). This parent felt that once they brought their children to occupational therapy, their child’s issues will be resolved. Consequently, parents felt that occupational therapists should develop their communication strategies to better explain the role of occupational therapy and adjust their use of language: “I think it could be pointed out to parents more … [tell them] we’re purposely looking for the areas [that the child has difficulty with]. And some sort of explanation around it or an audio visual would be even better part of interactive explanation but even a handout would be a good start” (Bill’s parent). Other parents spoke of the challenge in understanding jargon used by occupational therapists:

“Most of the parents have never heard of manual dexterity or sensory seeking or fine motor skills or any of that so when you’re handing a report back actually explain what it is and use layman’s terms” (Eddie’s parent).

In sum, this theme highlighted family’s experiences of services, and the negative impact of poor access to these services. Occupational therapists acknowledged their frustrations with long waiting lists. Ultimately, institutional barriers impacted family’s experiences of services and therapists’ capacity to work in ways they aspired to.

## Discussion

Perspectives of children, parents and occupational therapists were elicited to investigate experiences of children’s participation in decision-making in occupational therapy, which included examining the goal-setting process within the larger context of therapy intervention. All children in this study believed it was important to be listened to and to be respected. However, children recognised adults as power holders in decisions, acknowledging that they may have more knowledge than themselves, consistent with findings in Andersen and Dolva’s study [6]. Yet from the parent’s perspective, parents also did not experience being power holders and instead reported other factors relating to the broader context that impacted children’s participation in decision-making in goal-setting. Parents described stressful experiences of barriers to decision-making in accessing services and therapists described the stress of having to deliver services under extreme constraints of waiting list pressures.

The impact of waiting lists, lack of time and organizational pressures are frequently communicated when discussing public services in an Irish context [47]. Most parents in this study felt that it was necessary to ‘fight’ for public services to meet the needs of their children, a finding that has been illustrated in other studies [48, 49]. The limited nature of services for children and the reported challenges for parents in accessing such services has resulted in limiting meaningful participation opportunities for children. Critically, occupational therapists felt that these service limitations also impacted the development of rapport between occupational therapists and clients. Empowering children to participate in decisions that affect them is difficult when the adults themselves seemed disempowered by the systems that they work in. Hence, findings demonstrate that children’s voices can be subsumed by institutional agendas, priorities, and services, “situated within shifting power structures and relationships” [50, p. 41].

Within this context of power structures and relationships, children in this study had some positive and problematic experiences of decision-making in therapy. For example, although some children were happy to attend therapy, some were not but did not have a choice in making that decision. In addition, while half of the children (ages 6, 9 and 12) felt included in goal-setting, the other half (ages 8, 10, and 11) did not. Instead, child decision making was most evident in examples of events whereby children were offered a choice of picking an activity during therapy or in another example where a child chose not to take part in an activity, and the therapist supported his decision. In effect, decision-making was often relegated to involvement of children in these “smaller decisions” which is consistent with other studies of child participation in healthcare (for example, [3, 6]).

Documenting where participation of children in decision-making did not occur, seems somewhat easier to communicate than where participation occurred. For example, children and parents had unclear knowledge and information about what occupational therapy was about, and how it might help the child. Furthermore, assessment results seemed to be shared with parents and not with the children themselves (for example, a child living with anxiety as detailed in the findings) as is consistent with other studies in healthcare [5]. This is problematic given the fact that informed decision-making can only occur if a child and family have adequate information. Clearly questions remain; how much information should children be provided with, at what points in the therapy process should it be provided and how should it be communicated?

Despite decision-making being a right for all children, therapist’s commitment to children’s participation in decision-making was determined by their perception of the child’s level of competence combined with the lack of time to engage them in the decision-making process. Children less likely to be included in decision-making were those with communication difficulties, intellectual disability, or emotional and behavioral difficulties. Of note, while these children are deemed to have appropriate age and maturity from the perspective of Article 12 of the United Nations Convention on the Rights of the Child [1], they would require extra efforts to ensure decision-making was supported and their views taken into account. Evidence shows that a child can participate in decision-making once they are adequately supported [9] with evidence also showing that adults often lack competency in providing such support [51]. In this study, therapists identified strategies for engaging children who needed more support but acknowledged the lack of time to do so. Therefore, child participation in decision-making seemed more dependent on the lack of commitment of adults to the process, rather than the child or the therapist’s actual competency.

However, other factors were evident that influenced decision-making experiences for these children. Families in this study valued when occupational therapists made the child comfortable, were non-judgmental and were willing to listen and had an effective therapeutic relationship. Such factors have been found to support participation in decision-making [52, 53]. Yet, while most occupational therapists perceived that their goal setting was family-centred, some child and parent participants felt it took a more therapist-directed approach. This is consistent with the findings of Angeli et al. [54]. Parents confirmed that therapist decision-making takes priority, while therapists reported mainly giving priority to parent’s voice and working to balance expectations of parents. Notably, therapists considered that they played many roles to achieve this, including guiding, mediating, and orchestrating, highlighting the challenge in balancing a parent’s legal decision-making right with the child’s right to a voice in matters that affect them as set down in the United Nations Convention on the Rights of the Child [55]. However, this points to an imbalance within the triadic relationship within which negotiated decision-making occurs [55]. The nature of collaborative occupational therapy in pediatrics is generally a process between the (1) child, (2) parents and the (3) occupational therapist [24, 56]. Any imbalance in this triadic relationship may leave the child little space to be heard [57]. Ultimately, as noted in other studies, the adults remain the fundamental decision-makers, and it is adult-adult communication that determines goals and control decision-making for children in healthcare [3, 8, 58]. This evidence points to the prioritization given to family centered over child-centered practice in pediatric service provision and reflects a child-friendly rather than child-centered approach [14].

In this case, children’s voices are subsumed by the adult priorities, which, as noted earlier, are primarily concerned with the child’s best interests, and protection rather than participation [15, 21]. Children with disabilities can be viewed as needing protection, yet this is not typically the approach adopted in occupational therapy where autonomy, respect and choice is valued. Consequently, to address this need for a more balanced approach, occupational therapists can learn from advocates for a child’s rights-based approach to healthcare, who argue for the need to prioritize child-centred over family-centred practices in service provision [14].

The use of standardized tools to support effective goal-setting is one way to potentially address children’s decision-making. Goal-setting tools, such as the PEGS and the COPM have been successfully used in practice [59, 60]. Yet, therapists, children and parents in this study identified an informal approach in goal-setting without the use of standardized tools. Although informal goal-setting processes can be successful, they are slow to be adopted [61], difficult to implement [24], and do not ensure equitable opportunities for participation in decision-making [62]. What emerges from this study is the lack of formal processes in therapeutic practice, that is not limited simply to the goal-setting process but extends to the entirety of service delivery. The inconsistent processes that exist throughout occupational therapy services for children indicates a need to standardize implementation processes such as collaboration. Standardization offers the “opportunity to systematically evaluate the extent to which the processes are implemented and their impact on family satisfaction” [62, p. 45].

The United Nations Convention on the Rights of the Child [1] remains the most ratified convention in the world; nonetheless, therapists in this study were generally unaware of its significance to therapy practice. In the recent review of pediatric goal setting, a rights-based perspective of decision-making was not evident [63]. Instead, the prevalent theories that were identified included social cognitive and self-determination theories, health action process approach, mastery motivation and goal-setting theory [63]. While these serve to inform the knowledge base for pediatric goal setting, they omit the rights-based context which requires therapists to be defenders and enablers of a child’s rights in decision-making as an ethical issue of duty. Although the United Nations Convention on the Rights of the Child [1] clearly outlines the rights of a child’s participation in decision making, it’s utility as a meaningful framework that integrates a rights-based approach in occupational therapy practice may be limited by a lack of consensus in its interpretation [64]. Further exploration and clarity of concepts outlined in United Nations Convention on the Rights of the Child [1] is required such that its aspirations can translate meaningfully in child and family-centred practices [52].

### Limitations and future research

Several limitations should be considered when interpreting the results. Participants recruited to this study came from a small group of children who could communicate their ideas easily. Further research would be valuable with children who require support in communicating their views to enhance findings. Data collection was limited to one semi-structured interview for each participant; thus, the conclusions presented here potentially overlook perspectives that may have been derived from multiple interviews. Furthermore, the child-adult units interviewed as part of this study were not attending services provided by the occupational therapists potentially limiting the dependability or confirmability of the findings. However, it could be argued that there is a potential benefit to having not recruited triads as there was no potential threat to the therapeutic relationship. Future research should address these limitations and further examine children’s experiences of child-centredness in occupational therapy.

### Implications for occupational therapy policy, clinical practice and education

#### Policy and practice

As articulated by London [17] “institutional accountability for protecting human rights is essential to avoid shifting responsibility solely onto the health professional” (p. 65). Therefore, healthcare organizations are required to reduce contextual barriers that exist and create a milieu that supports children’s participation in decision-making within the goal-setting process. Development of policies and standards (in collaboration with children) in health and social care is crucial to creating a culture of participation in organizations where children’s participation becomes standard practice rather than a once-off activity [65]. Moreover, much work is required to determine the operationalization of Article 12 for the inclusion of the child’s voice in decision-making in the context of family-centred practice, while ensuring the protection of their best interests.

#### Education

Increasing human rights education in occupational therapy is necessary and aligns with the social change revisions to the Minimum Standard for the Education of Occupational Therapists [66]. Furthermore, the CRC [2] General Comment No. 12 has urged governments to provide child rights training for children and their parents, as well as for all professionals, including occupational therapists, who work with children.

## Conclusion

Findings from this study suggest that decision-making in goal-setting in occupational therapy is influenced significantly by the complexity of service delivery issues experienced by families. Critically, it seems that empowering adults to empower children is a major factor to facilitating children’s participation in decision-making. Although the findings in this study suggest occupational therapists always intend to include the views of children in goal-setting, parents and children reported this is not always the case. Although pediatric occupational therapists may work with vulnerable populations, there is a need to transform perspectives regarding the conceptualisation of children as being vulnerable to children being viewed as having agency and the competence to be involved in the decision-making process. Including children in decisions regarding goals by engaging in dialogue, knowledge transfer and ensuring space and time to express their views is one way to operationalize client-centred practice. Occupational therapists have an obligation and responsibility to engage with human rights [67]; thus, child-centred, right-based approaches are required to be central to pediatric occupational therapy, in line with the United Nations Convention on the Rights of the Child [1], United Nations Convention on the Rights of Persons with Disabilities [68], World Federation of Occupational Therapists [69], and the World Federation of Occupational Therapists Position Statement on Human Rights [70].

## Supporting information

S1 File Question guides and other methods. (DOCX)
