# Supplementary material for: A qualitative study of child participation in decision-making: Exploring rights-based approaches in pediatric occupational therapy
Source: PLoS One. 2021 Dec 16;16(12):e0260975. doi: 10.1371/journal.pone.0260975 (PMC8675724; doi:10.1371/journal.pone.0260975)
Supplement: S1 File — (DOCX) [file pone.0260975.s001.docx]

**Child Data Collection Guide**

*Introduction:*

Participants greeted, thanked for attendance.  An introduction would be given about the purpose and nature of the research study. Participant would be again made aware of their right to withdraw from the interview/overall study, confidentiality, and an overview of timespan also would be given. Children are given the opportunity to ask questions. Children can choose from these options:

1. *Role-Play*
2. *Photo elicitation*
3. *Drawing*
4. *Semi-structured interview*

*Details:*

1. *Role-Play*

Initiated by “Can you pretend that you are the OT”

1. *Photo elicitation*


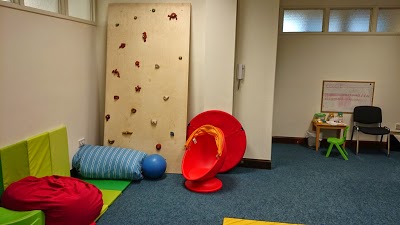


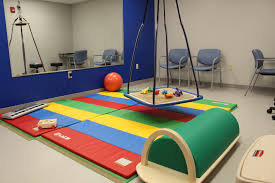


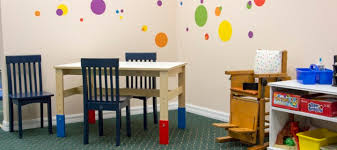


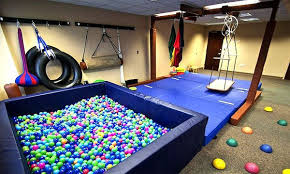


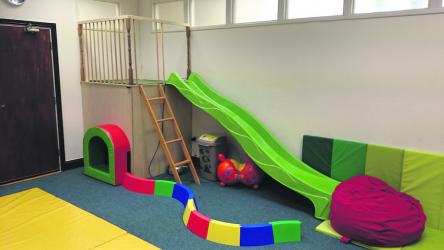


1. *Drawing*

Children would be asked to draw a session of occupational therapy from their experience

Name:

Age:

1. Semi-structured interview questions

General decision-making questions

Who decides what you do at school/home?

What decisions do children make at school/home?

Do you think children should be involved in making decisions?

What things might stop a child from being involved in decisions?

What helps a child to be involved in decisions?

__

Occupational Therapy specific questions

Tell me about going to Occupational Therapy

- What you did there?

Do you know why you were going to OT?

- If yes, who told you?

Did you make goals in OT?/ Did the OT ask you what you wanted to get better at/ do in OT?

-How?

-Who?

-Did you have a choice?

Did you do some of the things that you said you wanted to do?

- How did that make you feel?

Did you feel that the OT listened to you?

Did the OT talk to you about why your ideas were used/not used?

*End of interview:*

*Researchers thanks participant for their contribution. Reassure confidentiality, and anonymity. If any issues or queries have arisen as a result of this discussion, participants are encouraged to approach researchers or research supervisor. Participants will be given opportunity to review transcripts upon completion, and elaborate on subjects as they deem necessary. A certificate of appreciation would be given to children to thank them for their involvement.*

**Parent Interview Guide**

*Introduction:*

*Participant greeted and welcomed, thanked for attendance.  An introduction would be given about the purpose and nature of the research study.*

*Participant would be again made aware of their right to withdraw from the interview/ overall study, confidentiality, and an overview of timespan also would be given. Participants have the opportunity to ask questions.*

*Semi-structured interview questions:*

Tell me about _(child’s name)____

Tell me the things your child is involved in deciding at home?

Did your child know about why they were attending OT?

Do you think it is important to listen to children? Important that they are involved in decisions?

Tell me about what your child’s OT goals are/were

Do you think your child was asked about their goals/ included in decision-making process?

- do you think your child’s views should be included when picking OT goals? why/why not?

- did he/she do some of the things they identified as important to them in OT?

Tell me about your involvement in the goal setting process?

- was it important for you to be involved?

Were their things that helped your child having their voice heard in Occupational Therapy?

Are there things that might prevent your child’s voice being heard in OT?

*End of interview:*

*Researchers thanks participant for contributions. Reassure confidentiality, and anonymity. If any issues or queries have arisen as a result of this discussion, participants are encouraged to approach researchers or research supervisor. Participants will be given opportunity to review transcripts, upon completion, and elaborate on subjects as they deem necessary.*

**Occupational Therapist interview guide**

*Introduction:*

*Participant greeted and welcomed, thanked for attendance.  An introduction would be given about the purpose and nature of the research study.*

*Participants would be again made aware of their right to withdraw from the interview/overall study, confidentiality, and overview of timespan also would be given. Participants have the opportunity to ask questions.*

Tell me about the service that you work in

Would you consider your service to client/family-centred?

Tell me about how you approach informing a child about Occupational Therapy

Do you provide the child with relevant information about why they need to make goals?

Do you engage the child in goal setting?

If yes, in what way?

Tell me about your use of goal setting tools?

Are there specific policies in your service around child’s participation?

Tell me about if you think it is important for children to express their views in OT

Do you explicitly ask children about their goals? Why?

Is their opinion integrated into intervention?

Are children provided with feedback on why a decision has been taken?

Tell me about the factors that facilitate the child’s participation in the goal setting process

Tell me about the challenges to children’s participation in the goal setting process

*End of interview:*

*Researchers thanks participant for contributions. Reassure confidentiality, and anonymity. If any issues or queries have arisen as a result of this discussion, participants are encouraged to approach researchers or research supervisor. Participants will be given opportunity to review discussion transcripts, upon completion, and elaborate on subjects as they deem necessary.*
